# Impact of Orthodontic Appliances on Hiring Prospects in Saudi Arabia: A Cross-Sectional Study

**DOI:** 10.7759/cureus.40173

**Published:** 2023-06-09

**Authors:** Suliman Alsaeed, Kelvin I Afrashtehfar, Muneerah H Alharbi, Shaden S Alfarraj, Shahad A Alluhaydan, Fai A Abahussain, Ghaida M Alotaibi, Mohammed A Awawdeh

**Affiliations:** 1 Preventive Dental Sciences - College of Dentistry, King Saud Bin Abdulaziz University for Health Sciences, Riyadh, SAU; 2 Research Center, King Abdullah International Medical Research Center, Riyadh, SAU; 3 Dental Center, Ministry of the National Guard - Health Affairs, Riyadh, SAU; 4 Reconstructive Dentistry and Gerodontology, University of Bern, Bern, CHE; 5 Clinical Sciences Department, College of Dentistry, Ajman University, Ajman, ARE; 6 College of Dentistry, King Saud Bin Abdulaziz University for Health Sciences, Riyadh, SAU

**Keywords:** dental braces, clear aligners, orthodontics, cosmetic dentistry, dental aesthetics, malocclusion, esthetics, orthodontic appliances, personnel selection, employment

## Abstract

This study provides valuable insights into the cosmetic impact of orthodontic appliances on job-hiring prospects in Saudi Arabia. Both ceramic braces and clear aligners are considered cosmetic corrective devices compared to traditional metallic braces. This survey-based, cross-sectional study used two models, one male, and one female. Four standardized smiling frontal photographs were taken for each model: one without any appliance and three with different orthodontic appliances (i.e., metal braces, ceramic braces, and clear aligners). The photographs of each model were shown to potential employers, followed by three questions for each photo to assess the employers' views on the applicants' professionalism, communication skills, and the likelihood of being hired. The survey was distributed via an electronic questionnaire to employers in Saudi Arabia, collecting feedback from 189 participants. The sample was collected from October 2022 to February 2023. The models' scores while wearing metal and ceramic bracket appliances were significantly lower than when the models were wearing clear aligners or were not wearing any appliance in each domain. In conclusion, orthodontic appliances have cosmetic implications that affect job-hiring prospects, with a higher chance of being hired if the candidate does not have an orthodontic appliance.

## Introduction

Orthodontic appliances have undergone significant changes over time due to aesthetic demands. In the 1960s, stainless-steel brackets were introduced; lingual brackets were preferred until the 1980s, when tooth-colored ceramic brackets were introduced, and ceramic brackets and clear aligners are now in use [1-4]. However, there are significant advantages for metal brackets in certain cases over clear aligners due to their mechanical properties and tooth movement biomechanics [5-7]. This includes premolar-extraction cases that require bodily movements of molars and/or anterior teeth, which is challenging to achieve using clear aligners [8]. Also, cases with severe rotation can be easily corrected using metal braces in conjugation with coupling mechanics [9,10]. To overcome this limitation in clear aligners, a hybrid approach using metal braces in addition to clear aligners can be considered for optimum mechanical and aesthetic advantage [11,12].

The consideration of aesthetics in treatment planning has increased the demand and acceptance of orthodontic treatment by adult patients [13]. These patients prefer appliances with less metal showing and are less likely to accept unaesthetic treatments due to social, cultural, and personal factors [13]. Teeth are considered the most visible part of the face. Hence, a poor smile can affect an individual's social and psychological well-being [14]. Studies have evaluated the effects of orthodontic appliances on people's perception, including social competence, intellectual ability, psychological adjustment, and attractiveness [15-18]. Intellectual ability was associated with invisible lingual braces or no appliance [15]. The attractiveness of different appliance types was also evaluated [16]. The preference and acceptance of orthodontic appliances in the younger population, aged 9-17, have also been studied [17]. Clear aligners were the most popular appliances, followed by lingual and standard ceramic brackets. The least preferred appliance was hybrid brackets [18]. Age, sex, and culture can affect accepting different orthodontic appliances. Do orthodontic appliances affect employability potential? Studies have evaluated how orthodontic appliances affect the evaluation of an employee's professionalism [19]. Metal appliances were rated lowest in performance and attractiveness. However, orthodontic appliances did not affect the employees' overall evaluation. The dental appearance was not found to influence the decision to shortlist a candidate for a job interview or job performance, whether for public-facing or non-public-facing jobs [20]. However, the better the aesthetics of the appliance, the higher the chance of being hired. Clear aligners were preferred by young female evaluators while self-ligating aesthetic appliances were preferred by older female evaluators [21]. Male and female models showed statistically significant differences, with females having a higher chance of being hired.

The impact of orthodontic appliances on employment opportunities remains controversial in the literature. In Saudi Arabia, the demand for comprehensive orthodontic treatment is high, with reported rates reaching up to 77%, and significant variations in aesthetic and functional preferences [22]. It is well-established that perceptions of orthodontic appliance aesthetics vary across different societies and age groups. While studies have investigated such perceptions in the Saudi community, there remains a dearth of research on the potential impact of orthodontic appliances on job prospects. This study assesses the effects of various orthodontic appliances on the likelihood of being hired for jobs requiring personal interaction in Saudi Arabia.

## Materials and methods

**Figure 1:**
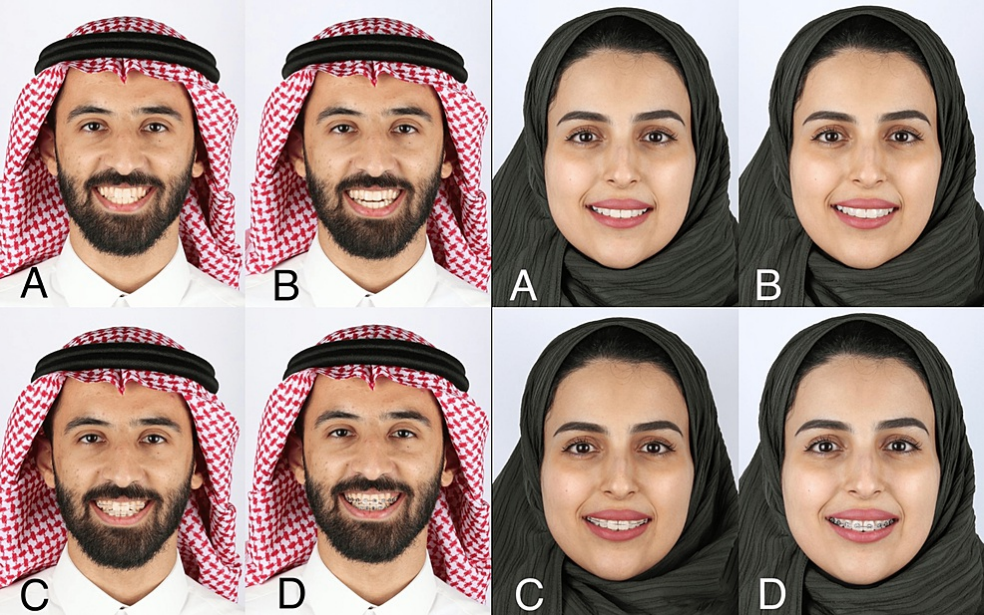
Frontal view of the male (left) and female (right) models in dynamic position or smiling with and without different orthodontic appliances. (A): No orthodontic appliance in place, (B): Presence of clear aligner, (C): A conventional ceramic appliance in place, (D): Presence of conventional metal appliance.

Ethical approval for this study was obtained from the Institutional Review Board at King Abdullah International Medical Research Center (KAIMRC) under protocol number SP21R/361/06. The study was conducted between October 2022 and February 2023 and the data was collected using an electronic questionnaire (Survey Monkey) in a survey-based, cross-sectional design directed by the human resources (HR) department's recruitment division. A simple random sampling technique was used to select participants. Two young adult models, one male and one female, with average smiles, were chosen to participate in the study. Three removable acrylic appliances were fabricated for each model using 0.018-inch steel wire based on the models' stone type IV or III diagnostic casts. The three removable appliances included conventional metal appliances, conventional ceramic appliances, and a clear aligner. Four standardized smiling frontal photographs were taken of the models wearing the traditional Saudi attire, three with the appliances and one without any appliances. The photos were taken using a Canon T5i Camera with the 60mm F2.8 Macro Ultra Sonic Motor lens and the Macro Ring 14EX Ring Flash. The photographs are displayed in Figure 1. The participants were recruited via email, social media, and in person. The participants were responsible for hiring and interviewing applicants for jobs that require personal customer interaction. Based on the Raosott sample size calculator, the sample size estimation for a population of 20,000 Human Resource individuals with a response distribution of 50% and margin error of 7.5% with 95% confidence interval was 170 participants.

The questionnaire was divided into three sections. The first section recorded the participant's demographic data and informed consent. The second section recorded the participant's general knowledge and exposure to orthodontic treatment. The third section included the four photographs of each model followed by three questions, and recorded using a visual analog scale (VAS) with 100 as the highest score. Participants were asked to rate the applicants' professionalism, communication skills, and likelihood of being hired. The standardized frontal photographs were designed to highlight the differences between orthodontic appliances, and each score assigned reflects the probability of this particular individual being hired. The VAS values were collected and recorded in an Excel sheet for statistical analysis.

Statistical analysis

The collected data were analyzed using statistical package software (IBM SPSS Statistics for Windows, Ver. 26.0; Armonk, NY: IBM Corp). Categorical data were presented as counts and percentages, while numerical data were presented as mean, standard deviation, median, and quartiles. The Wilcoxon signed-rank test was used to compare the ratings between the female and male models, while the Friedman test was used to compare the ratings of the four pictures of each model. All statistical tests were carried out at a significance level of 0.05.

## Results

There were 189 participants in this study, with a mean age of 31.1 ± 7.0 years. The majority of participants (67%) were men. Three-quarters of the participants held a bachelor's degree, and the participation was almost equally distributed across the five main regions of the Kingdom. About 35% of the participants had undergone orthodontic treatment, and 10% were currently undergoing treatment. Only one-third of the participants believed orthodontic appliances would affect a person's appearance (Table 1).

**Table 1:** Participants’ demographic characteristics.

		N	%
Age (years)	≤30	114	60.3%
	31+	75	39.7%
Highest education level	High school graduates or below	21	11.1%
	Bachelor’s degree	142	75.1%
	Postgrad degree	26	13.8%
Gender	Male	127	67.2%
	Female	62	32.8%
Region	Central	45	23.8%
	Eastern	36	19.0%
	Northern	35	18.5%
	Southern	37	19.6%
	Western	36	19.0%
Have you ever had an orthodontic treatment?	No	123	65.1%
	Yes, metallic brackets	61	32.3%
	Yes, clear aligner	4	2.1%
	Yes, ceramic brackets	1	0.5%
Are you currently undergoing orthodontic treatment?	No	170	89.9%
	Yes, metallic brackets	13	6.9%
	Yes, clear aligner	5	2.6%
	Yes, ceramic brackets	1	0.5%
Do you think that orthodontic appliances will affect a person’s appearance?	Yes	84	44.4%
	No	55	29.1%
	Maybe	50	26.5%

All participants rated the male model significantly lower than the female model in most domains and appliances, with three exceptions. First, there was no difference in rating between the two models when evaluating a person without an appliance for a job that requires face-to-face communication with customers. Second, there was no difference in rating for a person with a ceramic bracket being evaluated on their communication skills. Third, there was no difference in rating for a person with a ceramic bracket being evaluated on the level of their professionalism (Table 2). Similar results were observed when comparing the male model with the female model by male evaluators only (Table 3). However, the female evaluator had a different evaluation, with no significant differences in most cases. In most cases, the person had a metal bracket and was evaluated according to the three domains (communication skills, level of professionalism, and the chance of being hired). Additionally, when the person had a ceramic bracket, there was no significant difference in the level of professionalism and the chance of being hired (Table 4).

**Table 2:** Comparison of the ratings between male and female models in each domain and for each appliance by all participants. W, without any appliance; MB, metallic appliance; CB, ceramic bracket; AI, clear aligner; SD, standard deviation; M, Median; Q1, first quartile; Q3, third quartile; p-values were calculated using the Wilcoxon Signed Ranks Test. In all comparisons, the sum of negative ranks was less than positive ranks.

	Male Model		Female Model			
	Mean (SD)	M (Q1–Q3)	Mean (SD)	M (Q1–Q3)	Z	p−Value
W communication skills	78.6 (21.4)	81 (63–100)	84.3 (18.4)	90 (75–100)	−4.51	<0.001
W professionalism	78.5 (22.1)	84 (63–100)	83.2 (19.1)	89 (73–100)	−4.06	<0.001
W hired	81.1 (21.2)	89 (70–100)	84.0 (18.8)	90 (77–100)	−1.64	0.102
MB communication skills	72.9 (25.2)	79 (55–95)	75.1 (24.6)	80 (60–100)	−2.46	0.014
MB professionalism	73.9 (24.3)	80 (59–95)	75.5 (24.7)	81 (65–100)	−2.22	0.026
MB hired	69.0 (25.9)	75 (50–90)	72.5 (25.5)	80 (52–95)	−3.78	<0.001
CB communication skills	72.9 (23.2)	77 (55–90)	74.9 (23.5)	80 (59–99)	−1.90	0.058
CB professionalism	74.7 (22.1)	80 (60–91)	75.9 (23.0)	80 (63–100)	−1.71	0.088
CB hired	69.6 (25.0)	75 (52–90)	73.1 (23.8)	76 (55–95)	−3.30	0.001
AI communication skills	76.8 (22.4)	81 (61–100)	83.0 (19.7)	90 (75–100)	−5.61	<0.001
AI professionalism	77.9 (22.8)	85 (65–100)	83.3 (19.8)	90 (72–100)	−4.49	<0.001
AI hired	77.7 (22.8)	84 (64–100)	82.9 (20.2)	90 (74–100)	−4.17	<0.001

**Table 3:** Comparison of the ratings between male and female models in each domain and for each appliance by male evaluators. W, without any appliance; MB, metallic appliance; CB, ceramic bracket; AI, clear aligner; SD, standard deviation; M, Median; Q1, first quartile; Q3, third quartile; p-values were calculated using the Wilcoxon Signed Ranks Test. In all comparisons, the sum of negative ranks was less than positive ranks.

	Male Model		Female Model			
	Mean (SD)	M (Q1–Q3)	Mean (SD)	M (Q1–Q3)	Z	p−Value
W communication skills	77.9 (20.0)	80 (61–99)	82.6 (18.8)	87 (71–100)	−3.11	0.002
W professionalism	78.3 (21.8)	81 (65–100)	80.9 (19.9)	84 (70–100)	−2.17	0.030
W hired	79.6 (21.9)	87 (68–100)	81.3 (20.1)	85 (71–100)	−0.54	0.589
MB communication skills	73.2 (24.1)	77 (60–94)	74.7 (23.9)	80 (60–98)	−2.02	0.043
MB professionalism	74.2 (22.9)	80 (60–94)	75.1 (24.1)	81 (65–99)	−1.80	0.072
MB hired	68.7 (25.3)	73 (50–90)	72.7 (25.3)	79 (52–94)	−3.77	<0.001
CB communication skills	73.8 (22.0)	75 (59–90)	73.7 (23.3)	78 (59–93)	−0.05	0.964
CB professionalism	74.8 (21.8)	80 (63–91)	75.0 (22.8)	80 (64–95)	−0.70	0.484
CB hired	69.2 (24.5)	73 (52–90)	72.2 (24.0)	75 (54–91)	−2.72	0.007
AI communication skills	75.2 (23.4)	80 (56–100)	81.3 (20.6)	86 (73–100)	−4.29	<0.001
AI professionalism	75.8 (23.7)	81 (60–100)	81.8 (20.7)	88 (70–100)	−3.88	<0.001
AI hired	75.6 (23.8)	80 (62–100)	80.7 (21.3)	86 (70–100)	−3.61	<0.001

**Table 4:** Comparison of the ratings between male and female models in each domain and for each appliance by female evaluators. W, without any appliance; MB, metallic appliance; CB, ceramic bracket; AI, clear aligner; SD, standard deviation; M, Median; Q1, first quartile; Q3, third quartile; p-values were calculated using the Wilcoxon Signed Ranks Test. In all comparisons, the sum of negative ranks was less than positive ranks.

	Male Model		Female Model			
	Mean (SD)	M (Q1–Q3)	Mean (SD)	M (Q1–Q3)	Z	p−Value
W communication skills	80.1 (24.0)	90 (67–100)	87.6 (17.3)	97 (81–100)	−3.38	0.001
W professionalism	79.0 (22.9)	85 (62–100)	88.0 (16.3)	94.5 (82–100)	−4.14	<0.001
W hired	84.3 (19.4)	90 (74–100)	89.4 (14.6)	96.5 (85–100)	−2.04	0.041
MB communication skills	72.1 (27.7)	80.5 (50–100)	76.0 (26.0)	84.5 (51–100)	−1.48	0.138
MB professionalism	73.3 (27.3)	83.5 (53–100)	76.1 (26.1)	85 (56–100)	−1.32	0.186
MB hired	69.6 (27.4)	80.5 (49–92)	72.3 (26.1)	80 (52–100)	−1.20	0.231
CB communication skills	71.0 (25.6)	79 (50–91)	77.5 (23.9)	82 (60–100)	−2.90	0.004
CB professionalism	74.5 (23.0)	80 (54–93)	77.8 (23.5)	83.5 (62–100)	−1.81	0.070
CB hired	70.5 (26.0)	76 (50–91)	74.9 (23.5)	78.5 (58–100)	−1.80	0.072
AI communication skills	80.2 (19.8)	82 (70–100)	86.6 (17.2)	92 (80–100)	−3.62	<0.001
AI professionalism	82.0 (20.3)	88.5 (73–100)	86.4 (17.6)	93 (80–100)	−2.08	0.037
AI hired	81.9 (19.9)	90 (71–100)	87.3 (16.9)	93.5 (84–100)	−2.16	0.031

Young evaluators aged 30 years or less showed less differentiation between male and female models, mainly when the person was without an appliance and was being evaluated for their chance of being hired or when the person had metal brackets and was being evaluated according to the three domains (communication skills, level of professionalism, and the chance of being hired). There was no significant difference when the person had a ceramic bracket regarding their communication skills or professionalism (Table 5). However, in almost all cases, evaluators older than 30 years significantly rated male models lower than female models, except when the person was without an appliance and was being evaluated on their chance of being hired, and a person with a ceramic bracket being evaluated on the level of their professionalism (Table 6).

**Table 5:** Comparison of the ratings between male and female models in each domain and for each appliance by evaluators 30 years old or younger. W, without any appliance; MB, metallic appliance; CB, ceramic bracket; AI, clear aligner; SD, standard deviation; M, Median; Q1, first quartile; Q3, third quartile; p-values were calculated using the Wilcoxon Signed Ranks Test. In all comparisons, the sum of negative ranks was less than positive ranks.

	Male Model		Female Model			
	Mean (SD)	M (Q1–Q3)	Mean (SD)	M (Q1–Q3)	Z	p−Value
W communication skills	78.1 (21.9)	80 (60–100)	83.8 (19.3)	89.5 (75–100)	−3.73	<0.001
W professionalism	78.1 (23.3)	85 (62–100)	83.6 (19.2)	89 (70–100)	−3.33	0.001
W hired	80.5 (22.3)	89 (70–100)	84.3 (19.5)	90 (77–100)	−1.78	0.075
MB communication skills	72.4 (26.3)	78 (53–98)	73.3 (25.8)	79.5 (51–100)	−0.87	0.383
MB professionalism	73.9 (25.2)	81 (56–95)	74.4 (26.0)	81 (59–100)	−0.98	0.329
MB hired	68.8 (27.3)	75 (50–90)	71.5 (26.8)	76.5 (51–97)	−2.00	0.046
CB communication skills	71.7 (24.4)	75.5 (53–91)	73.3 (24.3)	79.5 (55–99)	−1.05	0.293
CB professionalism	72.8 (23.5)	79 (56–91)	74.9 (23.9)	80 (60–100)	−1.52	0.128
CB hired	68.6 (26.3)	75 (50–90)	71.9 (24.6)	75 (55–96)	−2.14	0.033
AI communication skills	76.7 (23.4)	80 (61–100)	83.5 (19.9)	90 (77–100)	−4.28	<0.001
AI professionalism	78.1 (23.3)	85 (65–100)	82.8 (20.9)	90 (70–100)	−2.97	0.003
AI hired	77.2 (23.4)	84 (62–100)	82.6 (21.6)	90 (74–100)	−3.03	0.002

**Table 6:** Comparison of the ratings between male and female models in each domain and for each appliance by evaluators older than 30 years of age. W, without any appliance; MB, metallic appliance; CB, ceramic bracket; AI, clear aligner; SD, standard deviation; M, Median; Q1, first quartile; Q3, third quartile; p-values were calculated using the Wilcoxon Signed Ranks Test. In all comparisons, the sum of negative ranks was less than positive ranks.

	Male Model		Female Model			
	Mean (SD)	M (Q1–Q3)	Mean (SD)	M (Q1–Q3)	Z	p−Value
W communication skills	79.3 (20.7)	82 (66–100)	85.0 (17.2)	91 (75–100)	−2.56	0.010
W professionalism	79.2 (20.4)	81 (64–100)	82.7 (19.0)	88 (73–100)	−2.51	0.012
W hired	82.1 (19.4)	89 (73–100)	83.5 (17.9)	90 (72–100)	−0.25	0.801
MB communication skills	73.6 (23.6)	80 (56–95)	77.9 (22.4)	83 (65–99)	−2.90	0.004
MB professionalism	74.0 (23.1)	77 (60–100)	77.1 (22.6)	83 (65–100)	−2.34	0.019
MB hired	69.3 (23.8)	73 (50–89)	74.1 (23.4)	80 (55–93)	−3.68	<0.001
CB communication skills	74.6 (21.3)	78 (58–90)	77.3 (22.1)	80 (70–96)	−1.97	0.049
CB professionalism	77.5 (19.7)	80 (64–100)	77.5 (21.7)	82 (65–100)	−0.85	0.396
CB hired	71.1 (22.8)	73 (53–91)	74.9 (22.7)	80 (53–95)	−2.65	0.008
AI communication skills	77.1 (20.9)	82 (60–97)	82.3 (19.4)	90 (73–100)	−3.65	<0.001
AI professionalism	77.5 (22.1)	81 (60–100)	84.1 (18.1)	90 (73–100)	−3.52	<0.001
AI hired	78.4 (21.8)	82 (64–100)	83.3 (17.9)	88 (74–100)	−2.79	0.005

Table 7 compares the ratings of different appliances for each model and each domain by all participants. In all cases, people with metal brackets and ceramic bracket appliances were rated significantly lower than when without any appliance or when they had a clear aligner.

**Table 7:** Comparison of ratings between different appliances for each model and each domain by all participants. W, without any appliance; MB, metallic appliance; CB, ceramic bracket; AI, clear aligner; SD, standard deviation; Q1, first quartile; Q3, third quartile; p-values were calculated using the Friedman test.

		Mean	SD	Median	Q1	Q3	p-Value
Male Model	W communication skills	78.6	21.4	81	63	100	<0.001
	MB communication skills	72.9	25.2	79	55	95	
	CB communication skills	72.9	23.2	77	55	90	
	AI communication skills	76.8	22.4	81	61	100	
	W professionalism	78.5	22.1	84	63	100	0.002
	MB professionalism	73.9	24.3	80	59	95	
	CB professionalism	74.7	22.1	80	60	91	
	AI professionalism	77.9	22.8	85	65	100	
	W hired	81.1	21.2	89	70	100	<0.001
	MB hired	69	25.9	75	50	90	
	CB hired	69.6	25	75	52	90	
	AI hired	77.7	22.8	84	64	100	
Female Model	W communication skills	84.3	18.4	90	75	100	<0.001
	MB communication skills	75.1	24.6	80	60	100	
	CB communication skills	74.9	23.5	80	59	99	
	AI communication skills	83	19.7	90	75	100	
	W professionalism	83.2	19.1	89	73	100	<0.001
	MB professionalism	75.5	24.7	81	65	100	
	CB professionalism	75.9	23	80	63	100	
	AI professionalism	83.3	19.8	90	72	100	
	W hired	84	18.8	90	77	100	<0.001
	MB hired	72.5	25.5	80	52	95	
	CB hired	73.1	23.8	76	55	95	
	AI hired	82.9	20.2	90	74	100	

## Discussion

This study aimed to explore the impact of orthodontic appliances on job opportunities in Saudi Arabia. Thus, we assessed the effects of different orthodontic appliances on hiring prospects and compared the perception of evaluators from both genders toward various orthodontic appliances worn by the models in our study. The evaluation of almost 200 participants evenly distributed across the five regions of Saudi Arabia revealed that the use of orthodontic appliances affected job opportunities for all evaluators. More than one-third of the sample had undergone orthodontic treatment. According to a study, people who have undergone orthodontic treatment often perceive themselves as less attractive while wearing orthodontic appliances [23]. This may explain why one-third of the participants think orthodontic appliances affect a person's appearance. 

Most participants were equal to or younger than 30 years old (60.3%), and the remaining 39.7% were older than 30. It is common for HR managers in Saudi Arabia to be younger than 30 years old [24]. Furthermore, most evaluators had a bachelor's degree (75.1%), followed by a postgraduate degree (13.8%), and a level of high school or below (11.1%). Most evaluators were male (67.2%), which may explain the biased result for female models. However, female evaluators also gave the female model significantly higher results than the male model. A study that measured the influence of orthodontic appliance design on employment hiring preferences found that the female model had higher results than the male model [13]. They attributed this result to women occupying more positions in public service and administrative sectors [13]. Participants were mainly distributed equally throughout the five regions (23.8% Central, 19.0% Eastern, 18.5% Northern, 19.6% Southern, and 19.0% Western). According to our results, female models had higher scores for all types of appliances, regardless of the evaluators' age and gender. Similarly, a 2019 study in Brazil by Didier et al. showed that all evaluators preferred the female model regardless of the appliance type [21]. The preference for women as employees might be because females hold more positions than men in public service and administrative divisions [25].

In most domains, male participants evaluated the female model higher than the male model, with a significant difference. In contrast, female participants showed no significant difference in most cases. However, they evaluated the female model with higher scores than the male model. Researchers have found that in more female-dominated occupations, there is discrimination against males; this may explain the high results given to female models by female evaluators [26,27]. Moreover, female evaluators gave much higher results for both male and female evaluators compared to male evaluators. When looking at the results, a significant difference was noted between the male and female models. For instance, the female model had higher scores than the male model. Respondents in the 30 years old or younger group had less significant results (Tables 5 and 6). Furthermore, the highest scores were attributed to the no-appliance pictures and the clear aligners, followed by ceramic and metal brackets, which had the lowest results overall.

Our findings reveal that the perception of orthodontic appliances varies among individuals. Research conducted by Ziuchkovski et al. has demonstrated that the attractiveness of different orthodontic appliances is perceived differently [16]. Notably, the Saudi population favors clear aligners as the most acceptable and aesthetically pleasing appliance [22]. This preference may be due to clear aligners' ability to provide a natural-looking appearance, which is supported by a study conducted by Didier et al. [21]. They found that clear aligners enhance smile aesthetics by making teeth appear shinier, and the open bite created by them improves the overall appearance. Our results are consistent with previous research that indicates judgments of others' personal characteristics are highly influenced by their dental appearance and smile design [28]. This also aligns with the systematic review conducted in 2020, which concluded that visible dental conditions could negatively impact assessments of employment-related personal characteristics [29]. Additionally, Pithon et al. found that individuals with ideal dental esthetics were perceived as more intelligent and more likely to find employment than individuals with nonideal smiles [30].

Our study's limitations should be considered when interpreting the results. Using static photographs may not fully represent the interview process and lacks the dynamic nature of a video. The photographs presented to participants were of near-ideal smiles, which may have skewed participants' perceptions of the appliances. Also, the study's sample size was relatively small, and participants were selected through convenience sampling. These limitations may limit the generalizability of the findings to other regions or populations. Additionally, the study did not assess the impact of different types of orthodontic appliances or consider other factors that may influence job hiring prospects. Future research should consider these variables.

Finally, it is worth noting that the study did not assess the long-term effects of orthodontic treatment on job hiring prospects. While the study found that the presence of orthodontic appliances had a negative impact on job hiring prospects, it is possible that the long-term benefits of improved dental aesthetics and confidence may outweigh the initial negative impact. Therefore, future research should investigate the long-term effects of orthodontic treatment on job hiring prospects to provide a complete picture of the relationship between dental aesthetics and employment outcomes.

## Conclusions

This study found that orthodontic appliances can significantly negatively impact job hiring prospects due to their cosmetic implications. Candidates wearing orthodontic appliances may be seen as less attractive and competent, leading to fewer chances of being hired. These findings highlight the importance of dental cosmetics in social judgments and hiring decisions. Individuals should be aware of the potential impact of orthodontic appliances on their professional life, and oral healthcare professionals should work with patients to minimize this impact.

Future research should investigate the mechanisms underlying this negative impact and identify strategies for mitigating it. Additionally, studying the long-term effects of orthodontic treatment on job hiring prospects and career success could help inform treatment decisions for individuals seeking cosmetic dentistry to improve their dental aesthetics.
